# Dissecting the Acid Stress Response of *Rhizobium tropici* CIAT 899

**DOI:** 10.3389/fmicb.2018.00846

**Published:** 2018-04-30

**Authors:** Julio Guerrero-Castro, Luis Lozano, Christian Sohlenkamp

**Affiliations:** ^1^Programa de Ecología Genómica, Centro de Ciencias Genómicas, Universidad Nacional Autónoma de México, Cuernavaca, Mexico; ^2^Programa de Doctorado en Ciencias Biomédicas, Centro de Ciencias Genómicas, Universidad Nacional Autónoma de México, Cuernavaca, Mexico; ^3^Programa de Genómica Evolutiva, Centro de Ciencias Genómicas, Universidad Nacional Autónoma de México, Cuernavaca, Mexico

**Keywords:** *Rhizobium tropici* CIAT899, pH, acid stress response, Tn*5*, transcriptome, RNA-Seq

## Abstract

*Rhizobium tropici* CIAT899 is a nodule-forming α-proteobacterium displaying intrinsic resistance to several abiotic stress conditions such as low pH and high temperatures, which are common in tropical environments. It is a good competitor for *Phaseolus vulgaris* (common bean) nodule occupancy at low pH values, however little is known about the genetic and physiological basis of the tolerance to acidic conditions. To identify genes in *R. tropici* involved in pH stress response we combined two different approaches: (1) A Tn*5* mutant library of *R. tropici* CIAT899 was screened and 26 acid-sensitive mutants were identified. For 17 of these mutants, the transposon insertion sites could be identified. (2) We also studied the transcriptomes of cells grown under different pH conditions using RNA-Seq. RNA was extracted from cells grown for several generations in minimal medium at 6.8 or 4.5 (adapted cells). In addition, we acid-shocked cells pre-grown at pH 6.8 for 45 min at pH 4.5. Of the 6,289 protein-coding genes annotated in the genome of *R. tropici* CIAT 899, 383 were differentially expressed under acidic conditions (pH 4.5) vs. control condition (pH 6.8). Three hundred and fifty one genes were induced and 32 genes were repressed; only 11 genes were induced upon acid shock. The acid stress response of *R. tropici* CIAT899 is versatile: we found genes encoding response regulators and membrane transporters, enzymes involved in amino acid and carbohydrate metabolism and proton extrusion, in addition to several hypothetical genes. Our findings enhance our understanding of the core genes that are important during the acid stress response in *R. tropici*.

## Introduction

The response to acidic stress conditions is understood best in enterobacteria, and *Escherichia coli, S. enterica* var. Typhimurium, *P. mirabilis* and *Y. enterocolitica* (Castanie-Cornet et al., 1999; Kieboom and Abee, 2006; De Biase and Pennacchietti, 2012), all have effective systems to contend with acid stress. Well-known are the decarboxylation systems, which are composed of two components: a decarboxylase and an antiporter. Protons are consumed in the cytoplasm through the decarboxylation of specific amino acids and the corresponding antiporter exports the decarboxylation product and imports more of the required amino acid (Foster, 2004). The best-studied example is the glutamate decarboxylase (Gad) system depending upon the concerted action of glutamate decarboxylase (GadA/GadB) and of the glutamate/GABA antiporter, GadC (Foster, 1999; Audia et al., 2001; Lund et al., 2014).

*Rhizobium tropici* CIAT899 is an α-proteobacterium capable of establishing a symbiosis with different leguminous plants including common bean (*Phaseolous vulgaris*) (Martínez-Romero et al., 1991). During this symbiosis root nodules are formed, which are specialized organs where biological nitrogen fixation (BNF) will take place (Suzaki et al., 2015). The efficiency of this symbiosis can be restrained by different environmental conditions, such as high temperature and low pH (Martínez-Romero et al., 1991; Graham et al., 1994; Hungría et al., 2000; Vinuesa et al., 2003). For example under acidic pH conditions, where survival and persistence of the bacteria are limited, nodulation and BNF can be severely affected. Acidic conditions can be found in the rhizosphere of plants, where the pH is lowered by plant exudates containing protons and organic acids, and inside symbiosomes (Udvardi and Day, 1997). Compared to most other nodule-forming bacteria *R. tropici* CIAT899 presents an increased resistance to acidic growth conditions.

A few studies trying to identify the genetic determinants of growth at acidic pH have been made in *R. tropici* (Riccillo et al., 2000; Vinuesa et al., 2003; Rojas-Jiménez et al., 2005; Vences-Guzmán et al., 2011). In 2003, Vinuesa et al. (2003), identified novel rhizobial genes required for acid tolerance. During a screen of a small Tn*5* mutant library composed of 1,728 clones, they identified two mutants affected in acid tolerance. In one mutant, the Tn*5* was inserted in the *sycA*-*olsC* gene cluster and in the second mutant, it was inserted in the *lpiA*-*atvA* operon. OlsC catalyzes the hydroxylation at the 2-position of the secondary fatty acid of ornithine lipids (OLs). The presence of this hydroxyl group has been correlated later to an increase tolerance to acidic conditions and high temperatures in CIAT899 (Vinuesa et al., 2003; Rojas-Jiménez et al., 2005; Vences-Guzmán et al., 2011). LpiA is a lysyl-phosphatidylglycerol synthase homologous to MprF from *Staphylococcus aureus* and the *atvA* gene is encoding a putative serine lipase homologous to the virulence proteins AcvB and VirJ from *Agrobacterium tumefaciens*. It was demonstrated that *lpiA* is induced under acidic conditions, and that LpiA participates in lysyl-phosphatidylglycerol (LPG) biosynthesis, which confers an increased resistance of *R. tropici* CIAT899 to the cationic peptide polymyxin under acidic growth conditions (Vinuesa et al., 2003; Sohlenkamp et al., 2007). Transcriptional induction of *lpiA* expression was also induced in *Sinorhizobium medicae* and *Sinorhizobium meliloti* as part of the response to low pH (Reeve et al., 2006; Hellweg et al., 2009). GshB participates in glutathione biosynthesis (Riccillo et al., 2000), which is necessary to grow in several environmental conditions like oxidative stress, osmotic stress and acid stress and transcription of *gshB* is induced under acidic stress conditions as shown by quantitative PCR (Muglia et al., 2007).

In addition, the CIAT899 genome encodes homologs to several proteins that are involved in acid stress response in other bacteria: (1) *eptA* encodes a putative lipid A phosphoethanolamine transferase, which confers acid resistance in *E. coli, Salmonella typhimurium*, and *Shigella flexneri* 2a (Martinić et al., 2011); (2) *cfa* encodes cyclopropane-fatty-acyl-phospholipid synthase which enhances acid tolerance in *E. coli* by reducing the permeability of the membrane to H^+^ (Shabala and Ross, 2008; Sohlenkamp, 2017). Five different copies of *cfa* genes are encoded in the genome of *R. tropici* CIAT899; (3) H^+^/Cl^−^ antiporters are responsible for protons extrusion when *E. coli* or *Helicobacter pylori* are exposed to acidic conditions (Inoue et al., 1999; Padan et al., 2004; Padan, 2008). CIAT899 possesses four genes encoding antiporters that possibly help to maintain pH homeostasis; (4) Exopolysaccharide (EPS) biosynthesis has been related to acid tolerance. In *S. meliloti* it has been shown that genes related to EPS production are expressed under acidic conditions (Cunningham and Munns, 1984; Hellweg et al., 2009) and the genome of CIAT899 encodes several *exo* genes and EPS production could help the bacteria to resist acid stress. It is not known, if the orthologues to these genes involved in acid stress response in other bacteria have a function during the acid stress response in *R. tropici*.

Vinuesa et al. (2003) had identified at least two novel genes involved in the acid stress response in *R. tropici* out of a screen using only 1,728 mutants. As the genome of *R. tropici* CIAT899 contains more than 6,280 genes (Ormeño-Orrillo et al., 2012), we think that it should be possible to discover further novel genes having a function in acid stress response in this organism. Here we present a study combining random transposon mutagenesis with transcriptomics to obtain a broad catalog of genes important or essential for the acid stress response in *R. tropici* CIAT899.

## Materials and methods

### Bacterial strains and culture conditions

Bacterial strains and plasmids used in this work are listed in Table 1. *R. tropici* CIAT899 was routinely grown in TY medium (Beringer, 1974) or the minimal medium described by Kingsley and Bohlool (Kingsley and Bohlool, 1992), adjusted to pH 6.8 [MM- buffered to pH 6.8 with 20 mM Hepes (*N*-(2-Hydroxyethyl)piperazine-*N*′-(2-ethanesulfonic acid))] or to pH 4.5 (MAM-buffered to pH 4.5 with 25 mM Homopipes (Homopiperazine-*N,N*′-bis-2-(ethanesulfonic acid))), Research Organics, Cleveland, OH, USA) at 30°C. MM and MAM were solidified with 0.8% gelrite (Carl Roth GmbH, Karlsruhe, Germany). *E. coli* strains were grown in Luria-Bertani (LB) medium at 37°C. Antibiotics were added at the following final concentrations (μg/mL): kanamycin (Km) 150; carbenicillin (Cb) 100; tetracycline (Tc) 10, and nalidixic acid (Nal) 20.

**Table 1:** Bacterial strains and plasmids used in this study.

**Strains or plasmid**	**Relevant characteristics**	**References**
***Rhizobium tropici***		
^A^CIAT899	A bean-nodulating strain acid tolerant, Nal^r^	Martínez-Romero et al., 1991
***Escherichia coli***		
DH5α	*recA*1, Δ*acU*169, _80d*lac*ZΔM1	Hanahan, 1983
HB101	*supE*44 *hsdS*20 (r–Bm–B) *recA*13 *ara*-14 *proA*2 *lacY*1 *galK*2 *rps*L20 *syl-5 mtl*-1	New England Biolabs
S17.1	*thi pro hsdR*– *hsdM*Δ *recA*, RP4 integrated in the chromosome, 2-Tc::Mu- Km::Tn*7*(Tp^r^/Sm^r^)	Simon et al., 1983
**PLASMIDS**		
pET17B	Expression vector, Cb^r^	Studier, 1991
pRK404	Broad-host-range vector, Tc^r^	Ditta et al., 1985
pBBR5MCS-2	Broad-host-range plasmid, Km^r^	Kovach et al., 1994
pUC18	Cloning vector, Amp^r^	Yanisch-Perron et al., 1985
pK18mobsacB	Conjugative suicide vector, Km^r^	Schäfer et al., 1994
pSUP1011	Mobilizable suicide plasmid for Tn*5* mutagenesis	Simon et al., 1983

*^A^CIAT stands for “Centro Internacional de Agricultura Tropical,” which is located in Cali, Colombia*.

### Tn*5* mutagenesis of *Rhizobium tropici* CIAT899

A general Tn*5* transposon mutagenesis of *R. tropici* CIAT899 was performed via conjugal transfer of pSUP1021 into CIAT899 utilizing *E. coli* S17.1 as the donor strain (Simon et al., 1983). Appropriate dilutions were plated on TY medium, supplemented with kanamycin and nalidixic acid to select for Tn*5* transconjugants. Individual colonies were transferred to microtiter plate wells with TY medium and were grown overnight at 30°C. Glycerol was added to a final concentration of 30% (w/v) and the mutant library was stored at −80 °C. To check the quality of the library, 10 transconjugants were randomly selected, genomic DNA was extracted, digested with *Eco*RI (New England Biolabs), and Southern blot hybridization was performed using a digoxigenin-labeled probe hybridizing to a fragment of the Tn*5*. The probe was synthesized using the oligos Tn5-1 and Tn5-2 (Table 2). This analysis established that the transposon had inserted in each strain only once and into different regions within the genome of *R. tropi*ci CIAT899.

**Table 2:** Primers used in this study.

**Primer**	**Length**	**DNA sequence (5′ to 3′)**	Tm^*^ (°C)	**References**
Tn5-1	20	CATTGAAGCGGGAAGGGACT	68.1	This study
Tn5-2	20	AGATCCTCGCCGTCGGGCAT	69.8	This study
BL	20	GGGGACCTTGCACAGATAGC	55.8	Huang et al., 2000
BR	23	CATTCCTGTAGCGGATGGAGATC	56.9	Huang et al., 2000
IR1	21	GAGCAGAAGTTATCATGAACG	50.3	Huang et al., 2000
IR2	29	CGGGATCCTCACATGGAAGTCAGATCCTG	64.3	Huang et al., 2000

Tm^*^ was calculated for DNA/DNA hybrid with a monovalent cation concentration of 100 mmol^−1^ using the program Tm calculator on the NEB webpage (http://tmcalculator.neb.com/#!/).

### Screen for acid-sensitive mutants

The ordered mutant library was replica-plated from the glycerol stock onto TY plates and grown for 3 days. Clones were transferred using a 48-pin replicator onto MM and MAM plates, and grown for 5 days. Mutants that grew as the wildtype under neutral pH conditions (MM), but that were strongly affected or that did not grow at low pH (MAM) were selected for further analysis. The Tn*5* insertion sites were mapped using two approaches. Most of the transposon insertion sites were determined using inverse PCR (iPCR) using the oligos described in Table 2. Briefly, 1 μg of genomic DNA of an acid-sensitive mutant is digested with the enzyme *Bam*HI (New England Biolabs) that cuts just in the middle of the Tn5. Following digestion, the reactions were inactivated at 65°C for 15 min. The restricted DNA was circularized overnight at 16°C in 100 μL reactions with 200 U of T4 DNA ligase (New England Biolabs), and then purified with High Pure PCR Product Purification Kit (Roche). The iPCR mix contained 10 μL of 10x PCR buffer, 6 μL of 25 mM MgCl_2_ (final concentration of 1.5 mM), 2 μL 10 mM dNTP mix (final concentration of 200 μM each dNTP), 0.5 μM (4 μL) of each primer (different combinations like BL-IR1, BL-IR2, or BR-IR1, BR-IR2 were used for the amplification, see Table 2), 10 μL (500 ng) of circularized template and 1.6 U of rTth DNA polymerase XL (Applied Biosystems) and ultrapure water (Milli-Q). The target DNA sequence was amplified using the following program: 94°C for 10 min, then 35 cycles 94°C for 1 min, 68°C for 5 min and 72°C for 10 min, and finally 72 °C for 10 min. PCR products were purified and sequenced (using different combinations of primers (see Table 2) at the Institute of Biotechnology (IBt) of the UNAM and mapped to the reference genome to identify the position of the Tn*5* insertions. For the other approach genomic DNA of acid-sensitive mutants was digested with *Eco*RI (cuts Tn*5* sequence without damaging the kanamycin resistance cassette) and then cloned into pUC19. After transformation we selected for kanamycin-resistant clones containing the resistance cassette and flanking sequences from the *R. tropici* genome. Plasmids were sequenced, and the insertion sites were mapped in the reference genome.

### RNA isolation

*R. tropici* CIAT899 was pre-grown in TY medium, then cultured in MM (control pH 6.8) (sample AR) or MAM (pH 4.5) (sample BR) until an OD620 of 0.6–0.7. For acid shock treatment cells were cultured in MM medium to mid-log phase, then washed with MAM and incubated another 45 min in MAM (pH 4.5) (sample CR). Cells were harvested by centrifugation and the pellets were immediately frozen in liquid nitrogen. Total RNA was prepared using the RNeasy mini kit (QIAGEN, Hildesheim, Germany) with some modifications. Pellets were resuspended in RLT buffer supplemented with lysozyme (20 mg per mL; QIAGEN, Hildesheim, Germany) and containing Zirconium Oxide Beads (0.5 mm, Next Advance). Cells were disrupted using the Bullet Blender Tissue Homogenizer (Next-Advance) in impact-resistant 2 mL tubes. Genomic DNA was eliminated by digestion with RNase-free DNase (QIAGEN, Hildesheim, Germany) for 20 min at room temperature. Final RNA concentrations were determined using a NanoDrop (Thermo Scientific). The typical OD260 to OD280 ratio of RNA samples was approximately 2.0. The integrity of RNA samples was verified using a TapeStation 2,200 instrument (Agilent Technologies) and the RNA integrity number (RIN^e^) was determined. Three independent total RNA extractions were obtained for each condition and each one was analyzed separately.

### RNA-seq and data analysis

RNA-Seq libraries were prepared using the TruSeq RNA sample Prep kit (Illumina) and sequenced (150 nt per read) by HiSeq 2500 instrument (Illumina) at the Beijing Genomics Institute (BGI, China). For the analysis of RNA-Seq data, Bowtie2 was used to align raw reads to the *R. tropici* CIAT 899 genome (Genbank entry CP004015, CP004016, CP004017, and CP004018) and samtools was used to obtain BAM files. Differentially expressed genes (DEGs) were obtained via NOISeq 2.14.1 Bioconductor package using local fit and betaPrior parameter set to False. NOISeq implements differential expression analysis based on the Negative Binomial distribution. A false discovery rate (FDR) threshold of 0.95 was set for DEG calling. Sample clustering and principal component analyses were performed upon variance stabilizing transformation of expression data (NOISeq package). Transcripts were called as differentially expressed when the FDR-Log2FC adjusted *p*-values were below 0.05 and fold-changes over 2 (Tarazona et al., 2011, 2015).

### Functional categorization of genes

The Clusters of Orthologous Groups (COGs) database was used to classify DEGsin *R. tropici* CIAT899 into functional categories (https://www.ncbi.nlm.nih.gov/COG/).

## Results

### Insertional mutagenesis of *Rhizobium tropici* CIAT899 and selection of acid-sensitive transposon mutants

**Figure 1:**
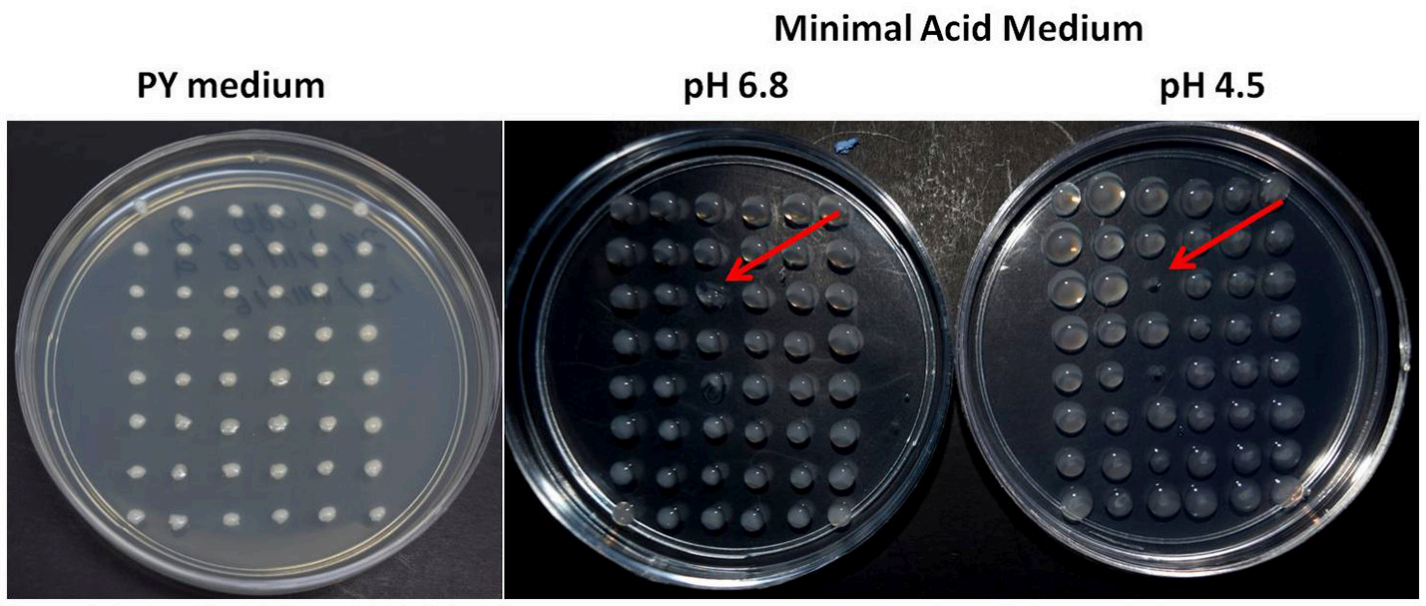
Screening of the *R. tropici* CIAT899 transposon mutant library for acid-sensitive mutants. A set of 48 Tn*5* mutants on complex TY medium, minimal medium (MM) and minimal acid medium (MAM) after 5 days of incubation. The arrow on the MAM plate indicates the position of an acid-sensitive strain (JG9587).

**Figure 2:**
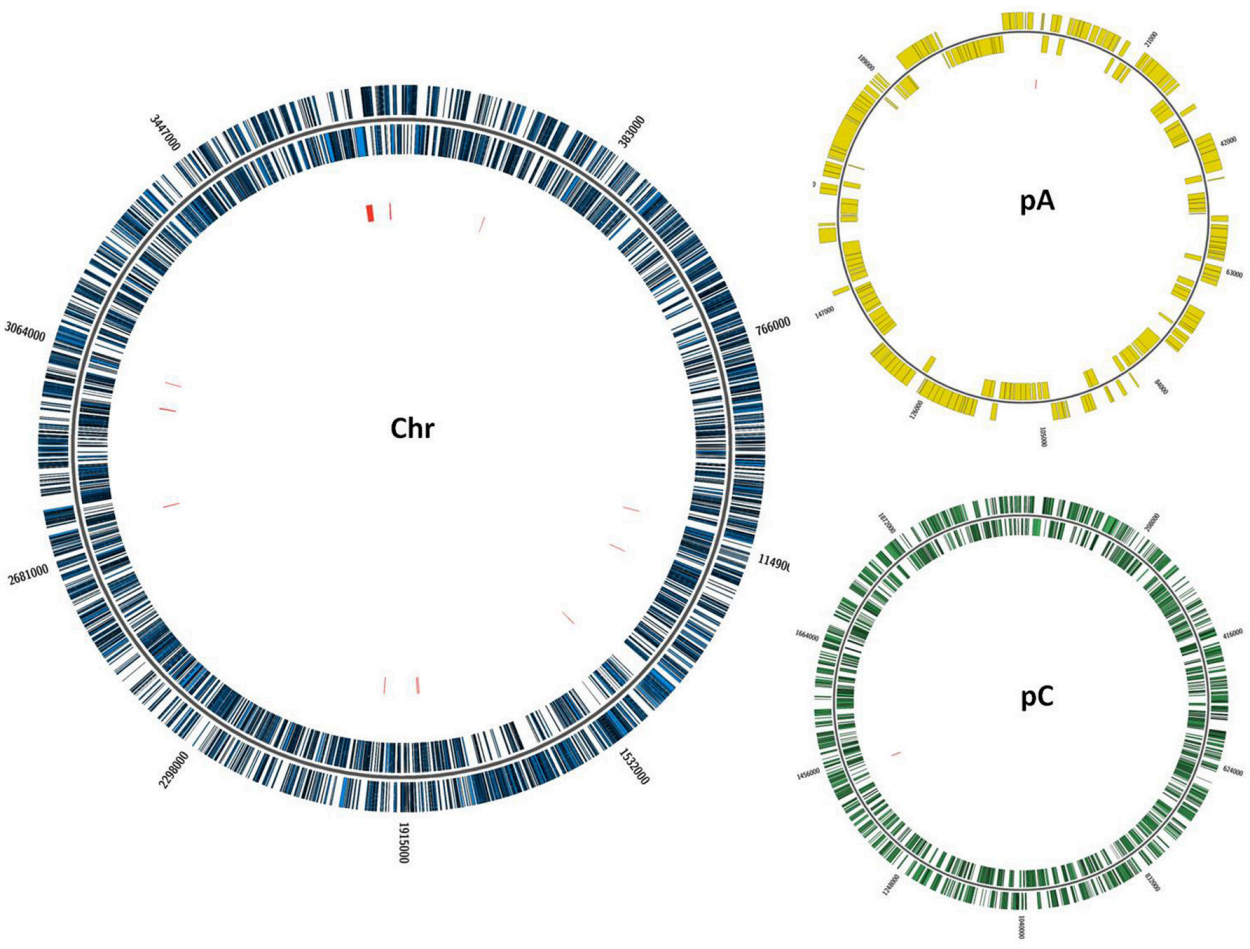
Location of transposon insertions (899::Tn*5*) in the *R. tropici* CIAT899 chromosome. Sites of transposon insertions are indicated by the red lines inside the circles. Colors in the three largest rings represent chromosome (blue), the smallest plasmid *pRtrCIAT899a* (pA) (yellow), and megaplasmid *pRtrCIAT899c* (pC) (green).

*R. tropici* CIAT899 has a 6.3 Mb genome in which some 6,280 protein-encoding genes are annotated. The aim was to saturate the genome with transposon insertions or at least to come close to saturation. An ordered library of 18,300 mutants was constructed using Tn*5*-derived transposons (Simon et al., 1983). For a small subset of mutants, we showed that each one presented a single Tn*5*-insertion and that they presented different RFLPs, indicating that the insertions were at different sites. Mutants were replica-plated on TY medium from the 96-well plate glycerol stock. After 3 days of growth, cells were replicated to minimal acid medium (MAM) and to neutral minimal medium (MM) to identify and select acid-sensitive mutants. Mutants were grown for 5 days, selecting those that grew at neutral pH as well as the wildtype but did not grow at acidic pH or that showed a drastically reduced growth under the latter condition (Figure 1). We grouped the mutants into two classes, those unable to grow at acid pH (AS: acid-sensitive) and those that presented only residual growth (MAS: mildly acid-sensitive). In total, 26 mutants were identified that showed growth deficiency under acid conditions, of these three are AS and 23 MAS at pH 4.5. We succeeded in identifying 17 Tn*5* insertions among these (Table 3). Most mutants presented insertions sites in the chromosome (14 mutants), except JG283 and JG9867, for which the insertion site was located in the megaplasmid pC (locus tag RTCIAT899_RS28660, polysaccharide biosynthesis protein). Megaplasmid pC has the characteristics of a chromid (Harrison et al., 2010), since it harbors essential genes involved in the biosynthesis of vitamins like thiamine and cobalamine. For mutant JG6057 the transposon insertion site was located to the plasmid pA (locus tag RTCIAT899_ RS18385, hypothetical protein). This is the smallest plasmid of *R. tropici* CIAT899, it is self-transmissible and includes various conjugation systems genes (Figure 2 and Table 3). None of the insertions was localized in the symbiotic plasmid (pSym or pB).

**Table 3:** Description of the acid-sensitive mutants identified in the mutant library.

**Mutant**	**Locus tag**	**Phenotype**	**log2FC**		**Gen product**
			**AR vs. BR**	**AR vs. CR**	
JG241	RTCIAT899_RS09205	MAS	0.082820403	0.358090297	Triose-phosphate isomerase
JG1042	RTCIAT899_RS09635	MAS	0.562135533	0.019664157	Ribulose phosphate 3-epimerase.
JG4882	RTCIAT899_RS05920	MAS	1.127623534	0.775841137	prolyl aminopeptidase
JG6187	RTCIAT899_RS18500	MAS	0.2711116	0.62190933	Acs acetyl-coenzyme A synthetase
JG5373	RTCIAT899_RS15005	MAS	−0.19186143	0.374167234	Glutathione-dependent formaldehyde-activating GFA
JG283 JG9867	RTCIAT899_RS28660	MAS	−1.16368769	−0.27188271	Polysaccharide biosynthesis protein (pC)
JG8656	RTCIAT899_RS13445	MAS	−0.16562978	−0.35630822	Ornithine lipid biosynthesis protein OlsC.
JG9587	RTCIAT899_RS14675	AS	0.033702161	−0.04398059	K+/H+ antiporter
JG163	RTCIAT899_RS14610	AS	0.810602571	0.312163145	Virulence factor family protein
JG9477	RTCIAT899_RS00995	MAS	−0.07145354	−0.56570741	MarR family transcriptional regulator
JG8654	RTCIAT899_RS09190	MAS	0.048965121	0.482194227	Transcriptional regulator FtrA
JG2646	RTCIAT899_RS05450	AS	1.131753457	1.39942044	DNA-binding response regulator
JG1076	RTCIAT899_RS31680	MAS	0.050745221	−0.56595384	Hypothetical protein
JG4655					
JG6057	RTCIAT899_RS18385	MAS	0.035556937	−0.30504672	Hypothetical protein (pA)
JG5634	RTCIAT899_RS20115	MAS	0.43645166	0.498978895	IS5/IS1182 family transposase

*The site of transposon insertion, the phenotype and the predicted gene product are described for each mutant. For comparison, the expression data obtained by RNA-Seq for these genes are presented. For example the transposon insertion in mutant JG2646 was localized to a gene encoding a DNA-binding response regulator and its expression was more than 2-fold induced in both cases (AR vs. BR and AR vs. CR). AR vs. BR = pH 6.8 vs. pH 4.5, AR vs. CR, pH 6.8 vs. acid shock for 45 min to pH 4.5, pA, pRtrCIAT899a; pC, pRtrCIAT899c*.

### Transcriptomic analysis shows that many genes are expressed differentially in response to acidic conditions

**Figure 3:**
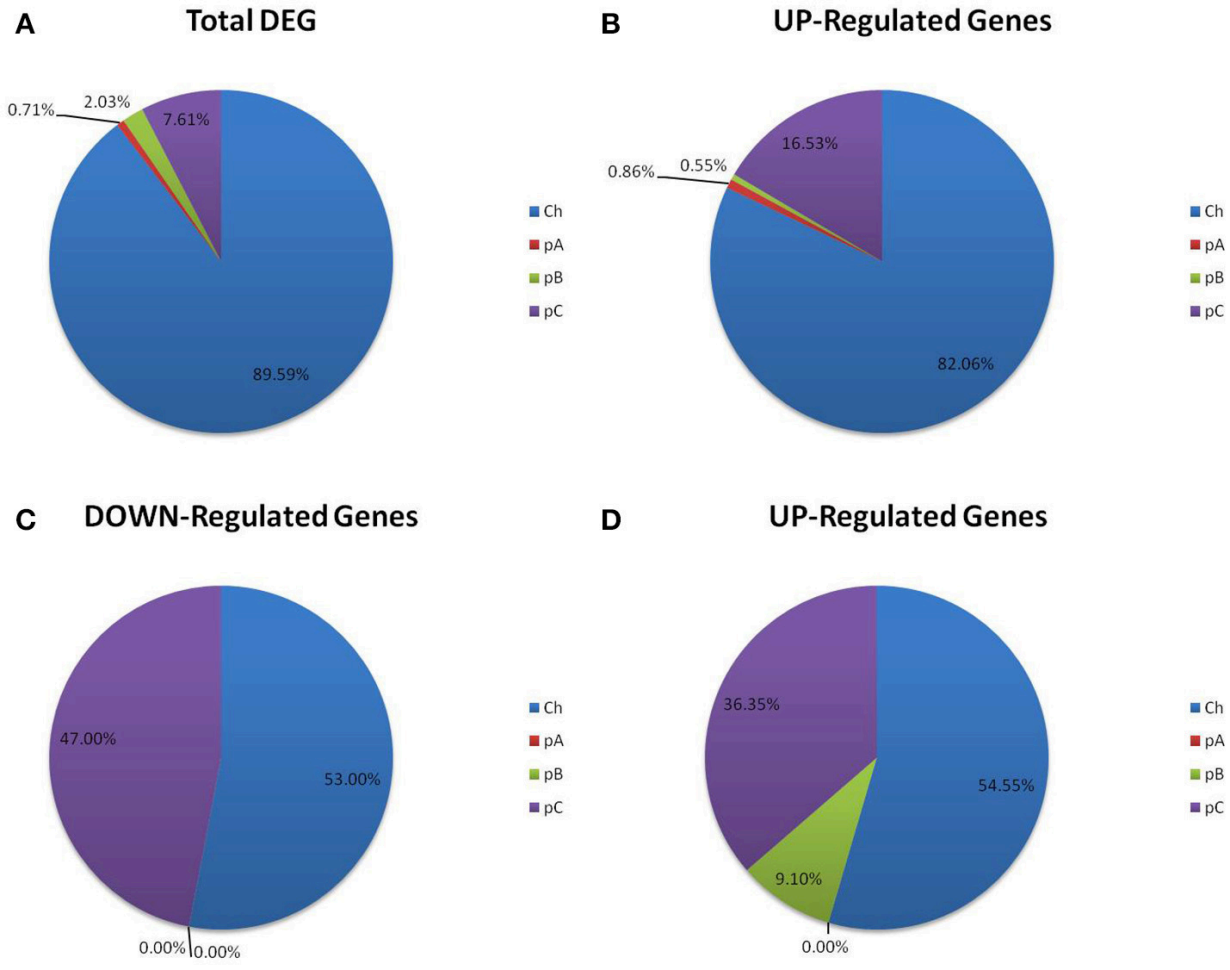
Distribution of differentially expressed genes (DEGs). Location of differentially expressed genes within the multipartite genome of *R. tropici* CIAT899. Total DEG **(A)**, up-regulated genes at pH 6.8 vs. pH 4.5 **(B)**, down-regulated genes at pH 6.8 vs. pH 4.5 **(C)** up-regulated genes at pH 6.8 vs. pH 4.5/acid shock **(D)**. Ch, chromosome; pA, pRtrCIAT899a; pB, pRtrCIAT899b; pC, pRtrCIAT899c.

The principal aim of this study was to identify novel genes important during acid stress in *R. tropici*. The screening of Tn5 mutants allowed us to identify genes that are important under acid stress, but dispensable under neutral pH conditions. Unfortunately, this approach would not allow for the identification of genes that are important during acid stress, but that are at the same time essential for growth at neutral pH. We guessed that these genes should be either transcriptionally induced or repressed during acid stress. *R. tropici* CIAT899 cells were grown under control conditions (pH 6.8 adapted, sample AR), in MAM at pH 4.5 (pH 4.5 adapted cells, sample BR), or pre-grown at pH 6.8 until an OD 600 nm of 0.6, washed and then transferred to pH 4.5 for 45 min (acid-shocked, sample CR). Three independent biological experiments were performed for each condition and transcriptomes were analyzed by RNA-Seq. Libraries were sequenced and between 6 and 11 million reads were obtained under each condition. Before subsequent analysis, a normalization process was carried out to eschew statistical deviations due to differences in library sizes (Alexandre et al., 2014). Differentially expressed genes in each condition were identified using the statistical software R (Figure 3). 394 genes were expressed differentially and most of these genes (353) were located in the chromosome, but a few were located in plasmid A (3), plasmid B (8), and plasmid C (30) (Figure 3A). RNA-seq data were submitted to the Sequence Read Archive (SRA) database with the accession number GSE108074.

**Figure 4:**
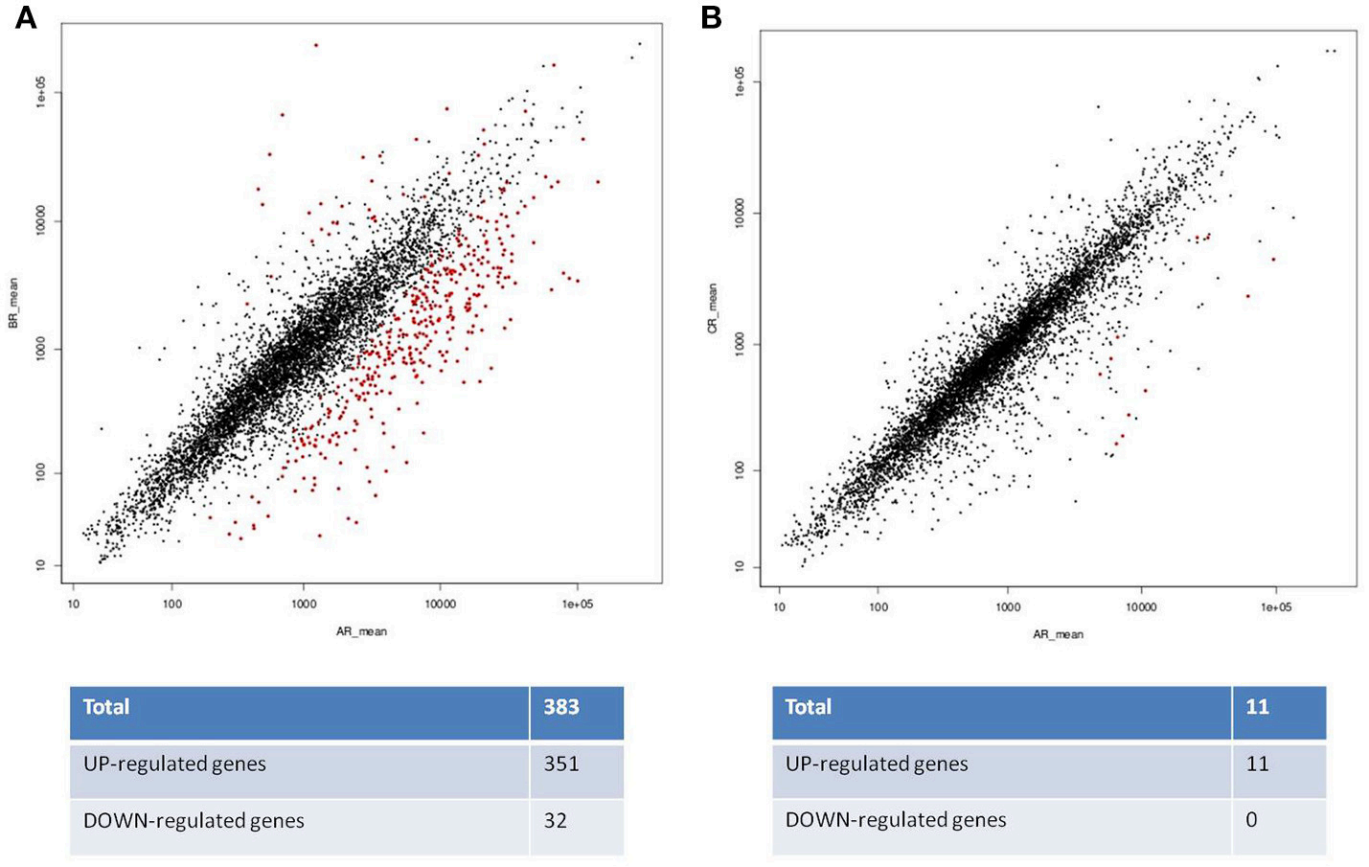
Summary plot of the expression values for both comparisons. Each dot corresponds to an expressed gene, differentially expressed genes are highlighted in red. **(A)** DEG pH6.8 (AR), vs. pH 4.5 (BR) and **(B)** DEG pH6.8 (AR) vs. 45 min acid shock at pH 4.5 (CR).

When comparing the transcriptomes of cells grown at pH 6.8 to cells adapted to pH 4.5, we observed that 351 genes were induced (chromosome: 288, pA: 3, pB: 2 and pC:58, respectively) (Figure 4A). The large majority of these genes was located on the chromosome and several others on plasmid C. Only very few of the induced genes were on plasmids A and B. 32 genes were repressed (chromosome: 17 and pC: 15) (Figures 3B,C, 4A). However, when comparing the transcriptomes of cells grown at pH 6.8 to the transcriptome of cells pre-grown at pH 6.8 and then acid-shocked for 45 min, only 11 genes were up-regulated (chromosome: 6, pB: 1 and pC: 4) and no gene was down-regulated under these conditions (Figures 3D, 4).

### Functional analysis of differentially expressed genes

**Figure 5:**
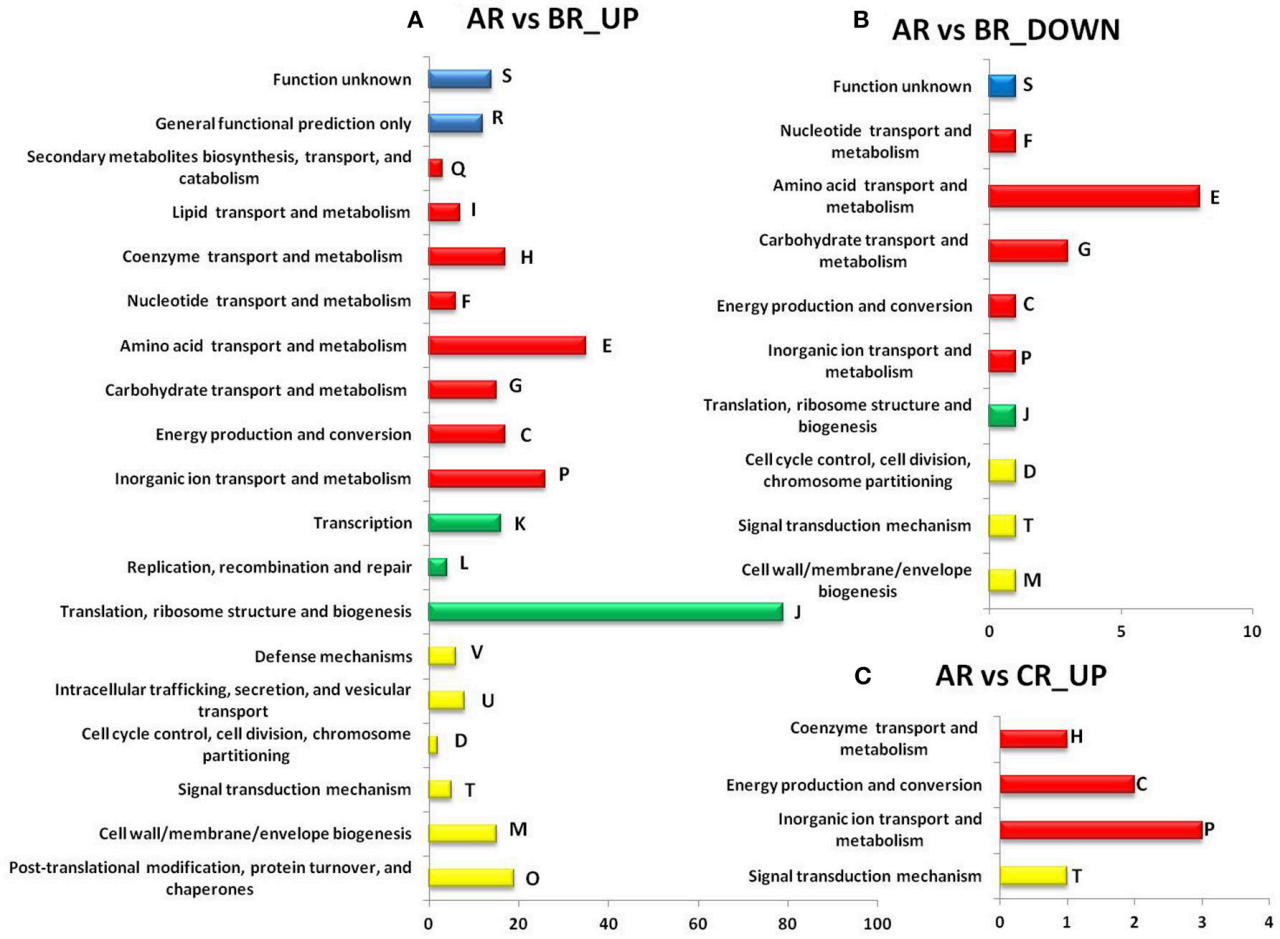
Differentially expressed genes classified in COGs (Clusters of Orthologous Groups) categories. COGs of genes up-regulated **(A)** and down-regulated **(B)** genes and COGs up-regulated affected after acid shock **(C)**. General category letter associations by groups and letters (see above); in yellow: cellular processes and signaling (D, M, O, T, U, V), in green: information storage and processing (J, K, L), in red: metabolism (C, E, F, G, H, I, P, Q) and blue: poorly characterized (R and S) (Tatusov et al., 2000, see NCBI COG website). The X axis shows the number of DEGs.

Genes that were differentially expressed (DEGs) were grouped according to the COGs protein database (https://www.ncbi.nlm.nih.gov/COG/) and were classified into four principal categories: (1) processes and signaling, (2) information storage and processing, (3) metabolism and (4) poorly characterized (Figure 5). DEGs assigned to these principal categories were further sub-grouped. When comparing the transcriptomes of acid-adapted cells (sample BR) and control cells (sample AR), of the 126 differently expressed genes grouped into the COG category metabolism, 35 DEGs were assigned to amino acid transport and metabolism (E), 26 DEGs to inorganic ion transport and metabolism (P), 17 DEGs to energy production and conversion (C) and to coenzyme transport and metabolism (H), 15 DEGs to carbohydrate transport and metabolism (G), 7 DEGs to lipid transport and metabolism (I), 6 DEGs to nucleotide transport and metabolism (F) and finally 3 DEGs were assigned to the sub-category secondary metabolites biosynthesis, transport, and catabolism (Q). Another principal category that is also over-represented is information storage and processing (99 DEGs). Of these DEGs, 79 DEGs were grouped to the sub-categories translation, ribosome structure and biogenesis (J), 16 DEGs to transcription (K) and 4 DEGs to replication, recombination and repair (L). The third principal category is cellular processes and signaling with 55 DEGs. Of these, 19 DEGs were assigned to post-translational modification, protein turnover, and chaperones (O), 15 DEGs to cell wall/membrane/envelope biogenesis (M), 8 DEGs to intracellular trafficking, secretion, and vesicular transport (U), 6 DEGs to defense mechanisms (V), 5 DEGs to signal transduction mechanism (T) and 2 DEG to cell cycle control, cell division, chromosome partitioning (D). Finally, a few DEGs are poorly characterized and cannot be classified within the other principal categories. This includes General functional prediction only (R, 12 DEGs) and Function unknown (S, 14 DEGs) (Figure 5A). When we analyzed the DEGs that were repressed comparing the transcriptomes of acid-adapted cells and control cells, 8 DEGs were grouped in amino acid transport and metabolism (E), and 3 DEGs in carbohydrate transport and metabolism (G). To a few other subcategories, one DEG each was assigned (M, T, D, J, P, C, F, and S). When comparing the transcriptomes of acid-shocked cells and control cells, we found only 11 DEGs, with 6 DEGs grouped into the principal category metabolism and one DEG grouped into the principal category information storage and processing (Figure 5C). In total, 62 DEGs could not be classified in COGs (for all conditions).

## Discussion

The effects of acid stress have been characterized best in *Enterobacteriaceae* that can be exposed to extreme changes in extracellular pH as they pass through the inhospitable environment of the stomach into the lower digestive tract (Foster, 2004; Lund et al., 2014). Rhizobia show significant variability in their ability to grow under different low pH conditions, and within this family *R. tropici* CIAT899 is among the most tolerant strains (Martínez-Romero et al., 1991; Graham et al., 1994). The capacity to grow and persist under environmental changes is essential for survival in the rhizosphere and within the nodules of leguminous plants. A Tn*5*-based transposon library of 18,300 insertional mutants was screened to identify mutants growing as the wildtype at neutral pH, but that did not grow or that showed a drastically reduced growth under low pH conditions. This strategy aimed at identifying genes specifically required for the acid stress response, but not for growth at neutral pH. We isolated 26 Tn*5* mutants that were unable to grow under acidic conditions (see Table 3). For 17 of these we could identify the transposon insertion sites, which could be located mainly to the chromosome, but also to the plasmids pA and pC. None of the selected mutants presented an insertion site in the symbiotic plasmid pB (Figure 2).

Vinuesa et al. (2003) had made a similar screening, but on a much smaller scale. One of the mutants identified in the earlier study had an insertion in the *sycA/olsC* gene cluster (Vinuesa et al., 2003; Rojas-Jiménez et al., 2005). Interestingly, the mutant JG8656 identified in the present study also had an insertion in the gene *olsC*. This gene codes for the ornithine lipid (OLs) hydroxylase OlsC, which is responsible for the 2-hydroxylation of the secondary fatty acid in OLs. This hydroxylation has been correlated to an increased resistance of *R. tropici* to acid stress and high temperatures (Vences-Guzmán et al., 2011). One hypothesis is that the presence of the additional hydroxyl group allows the formation of hydrogen bonds between lipid head groups, thereby making the membrane less fluid and less permeable to protons (Nikaido, 2003; Rojas-Jiménez et al., 2005; Vences-Guzmán et al., 2011; Sohlenkamp and Geiger, 2016). Another gene found by Vinuesa et al. was *lpiA* (*l*ow *p*H *i*nducible) encoding a putative lysyl-phosphatidylglycerol (LPG) synthase, implicated in LPG biosynthesis. LPG is a membrane lipid whose presence confers resistance to various cationic peptides to *S. aureus* (Peschel et al., 2001). The *lpiA* gene is found in an operon with *atvA* (*a*cid *t*olerance and *v*irulence), which is an orthologue of *acvB* from *A. tumefaciens*. Interestingly, in our study we found a mutant (JG163) affected in *atvA*, which is consistent with the results obtained by Vinuesa and co-workers (Vinuesa et al., 2003). Furthermore, three mutants were affected in a gene whose product is a hypothetical protein (JG1076, JG4655 and JG6057), in this case two of them have an insertion in the same ORF (JG1076, JG4655). Interestingly, two more mutants (JG283 and JG9867) also presented transposon insertions in an identical ORF. This could be indicating that the transposon mutagenesis is close to saturation.

Mutant JG241 presents an insertion in a gene encoding a putative triose phosphate isomerase enzyme (*tpiA1*). It has been reported that *H. pylori*, in addition to the genes involved in urea hydrolysis, induces genes at pH 5.5 that are related to carbohydrate metabolism (including the *tpiA* gene). An explanation suggested was that the cell's energy requirement is increased under this condition (Ang et al., 2001; Wen et al., 2003; Zanotti and Cendron, 2010). Besides, we found other mutants with insertions in genes related to anabolic and energy generation process like ribulose phosphate 3-epimerase (locus tag RTCIAT899_RS09635), prolyl aminopeptidase (RTCIAT899_RS05920) and acetyl-coenzyme A synthetase (Acs) (RTCIAT899_RS18500). Another mutant (JG283) presented an insertion in a gene encoding a polysaccharide biosynthesis protein. Polysaccharide and exopolysaccharide biosynthesis has been reported to help counteract high proton concentrations by preventing protons from passing into the cell (Aarons and Graham, 1991; Graham et al., 1994; Reeve et al., 1997, 1998; Hellweg et al., 2009). Mutant JG9587 has a Tn*5* insertion in an ORF encoding a putative antiporter protein, whose function could be to expel protons toward the periplasm under acid stress conditions. In *E. coli* the Na^+^/H^+^ antiporter (NhaA) helps to maintain Na^+^ and H^+^ homeostasis and several reports indicate that NhaA activity is increased at high or neutral pH (Padan et al., 2004; Padan, 2008). In contrast, *H. pylori* has a Na^+^/H^+^ antiporter whose activity is high at acidic and neutral pH, indicating that *H. pylori* employs this antiporter under acidic conditions (Inoue et al., 1999).

In some bacteria an increase of the extracellular proton concentration is sensed through two-component systems (Lund et al., 2014), examples being the PhoPQ system from *S. typhimurium* which is induced in acidic conditions (Prost et al., 2007) and the ArsRS system from *H. pylori*, which senses increased proton concentrations and responds by regulating genes of biosynthesis and metabolism of the urease system (Pflock et al., 2006). Mutant JG2646 presented a transposon insertion in a gene encoding a response regulator. This gene is found in an operon with a gene encoding a histidine kinase, and so this two-component system may be sensing the external pH and may be responsible for the activation of a subset of genes under this condition. Two more mutants had transposon insertions in genes encoding transcription factors (JG9477 and JG8654). These genes code for proteins with homology to MarR and FtrA (locus tag RTCIAT899_ RS00995 and RTCIAT899_RS009190), respectively. MarR presents similarity to the transcriptional regulator Rv1404 from *Mycobacterium tuberculosis*, which regulates the transcription of the genes *rv1403c* and *rv1405c* encoding putative *S*-adenosyl methionine (SAM)-dependent methyltransferases. This activation under acid stress conditions (pH 5.5) also requires *phoP* (Healy et al., 2016). In the case of FtrA, there are no reports about its role acid stress conditions.

In mutant JG5373 the transposon insertion is probably affecting a gene whose product might be involved in catalyzing the condensation of formaldehyde and glutathione to *S*-hydroxymethylglutathion (*gshA*). In the genomic context of *R. tropici* CIAT899, a glutamate synthase (possibly *gshB*) is located downstream of *gshA*. Ricillo et al. reported that a mutant affected in a gene involved in the biosynthesis of tripeptide glutathione (*gshB*) in *R. tropici* was affected in growth under acidic conditions (Riccillo et al., 2000). It is possible that the transposon insertion in the mutant JG5373 creates a polar effect affecting glutathione biosynthesis and causing susceptibility to acid stress. Most often an polar effect occurs when the transposon is inserted into the first ORFs of an operon affecting the expression of the downstream genes of the same operon (Zipser, 1969). In our study, we identified three mutants (JG4882, JG163 and JG2646), whose Tn*5* insertion occurred in putative operon, however, only the mutant JG2646 has the insertion in the first gene (Supplementary Figure S1) of the operon. The latter transposon insertion can be expected to cause a polar effect on the expression of the downstream genes. This hypothesis was verified by a complementation assay of mutant JG2646: the gene encoding for the response regulator alone was not able to complement the mutant phenotype when provided in trans whereas the complete operon (including the histidine kinase (HK), locus tag RT899_RS05455) complemented the mutant phenotype when provided in trans.

**Figure 6:**
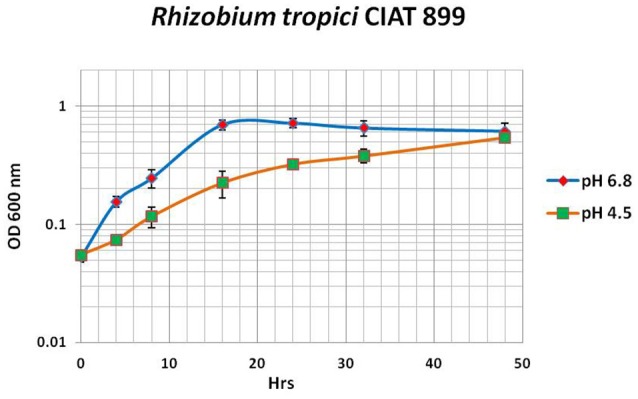
Growth of *Rhizobium tropici* CIAT 899 in minimal medium adjusted to different pH values. Wild type was grown either in MM (pH 6.8, blue) or MAM (pH 4.5, red). Cultures were grown at 30°C in a gyratory shaker. Growth was observed by measuring the OD 620 and the generation time was calculated (2.2 h at pH 6.8 and 1.9 h at pH 4.5). Mean values for triplicate replicates are shown for each condition ± standard deviation (bars).

The results obtained by our library screening indicate that outside of certain conserved components of the acid stress response, such as response regulators or antiporters, the genes necessary for an acid stress response vary from species to species. To study the acid stress response from another angle we used RNA-seq to analyze the transcriptomes of *R. tropici* CIAT899 cells grown at neutral pH (6.8, control condition), of cells grown at pH 4.5 (acid-adapted cells), and of cells pre-grown at pH 6.8 and then acid-shocked for 45 min at pH 4.5. We chose such a short time (45 min) for the acid shock because we were interested in the very first transcriptional responses. Earlier transcriptome studies in *S. meliloti* 1021 applied a pH shift from pH 7.0 to pH 5.75 (the lowest pH at which *S. meliloti* grows) for varying times (from 3 to 63 min), observing the maximum induction between 33 and 63 min (Hellweg et al., 2009). Our RNA-seq results exhibit that gene expression of *R. tropici* CIAT899 was broadly changed by the acid stress. Hundreds of genes (351 up-regulated and 32 down-regulated in acidic conditions and 11 up-regulated in acid shock) were significantly up- or down-regulated under acidic conditions (Table 4 and Supplementary Table S1). These differently expressed genes (DEGs) can be functionally grouped according to the predicted functions of the encoded proteins. We identified several genes associated with metal transport (like RTCIAT899_RS04615, RTCIAT899_RS04620, RTCIAT899_RS04610, RTCIAT899_RS17535, RTCIAT899_RS10385, or RTCIAT899_RS14255). It is known that in tropical acid soils high concentrations of metals like Zn^2+^, Co^2+^, Cd^2+^, Ni^2+^, and often, Mn^2+^, Fe^2+^, Cu^2+^ and mercury ions are very common. Therefore, these metal transporters could be contributing to the efflux of these metals to avoid the toxic effects of metal ions (Montanini et al., 2007) when the cells encounter high proton and metal concentrations. ABC transporters transport solutes across the membrane utilizing the energy of ATP hydrolysis and they have been over-expressed after acid shock in other bacteria, including *S. aureus* (Bore et al., 2007). In our study, several of the identified DEGs encoded ABC transporters that were significantly over-expressed under acid stress (Jia et al., 2017) (Figure 5 and Supplementary Table S1). Furthermore, we identified a few putative transcription factors that might be implicated in the regulation of efflux pumps under acid stress (like DeoR, RTCIAT899_RS29995; TetR, RTCIAT899_RS10760, RTCIAT899_RS28830, or YebC/PmpR, RTCIAT899_RS14585). The TetR family of transcriptional regulators has been reported to be involved in the regulation of efflux pumps (Perrone et al., 2017) and drug efflux in mycobacteria (Betts et al., 2003; Wei et al., 2014). Among the DEGs that were up-regulated were also the *hyc* genes encoding hydrogenases proteins. In *E. coli* and *Salmonella* these hydrogenases can reduce acid stress by proton consumption and H_2_ production. In this study, 10 genes encoding hydrogenases were over-expressed and the transcriptional induction of hydrogenase genes observed in this study is probably involved in the acid stress response, indicating that *R. tropici* uses this mechanism to resist high proton concentrations (Supplementary Table S1; Hayes et al., 2006; Zbell and Maier, 2009; Noguchi et al., 2010; Jia et al., 2017). F_0_F_1_ synthase normally catalyzes the synthesis of ATP from ADP using the energy derived from an electrochemical proton gradient. Under acid stress conditions, hydrolysis of ATP may be used to expel protons from the cytoplasm. This flow of protons through the F_0_ subunit could contribute to pH homeostasis and therefore contributed to acid stress response like in *E. coli* or *Corynebacterium glutamicum* (Diez et al., 2004; Barriuso-Iglesias et al., 2013). In our study we identify two gene clusters encoding several subunits of F_0_F_1_ ATPase encompassing nine genes involved in the assembly of the two units of ATPase (F_0_ and F_1_), which are up-regulated more than 4 times at pH 4.5 compared to pH 6.8. Sigma factors are subunits of the bacterial RNA polymerase (RNAP) playing important roles in transcription initiation, especially during promoter recognition. Specific sigma factors have functions during the differential expression of genes during abiotic stress, during development or during specific growth phases (Paget, 2015). The sigma factor E (SigE) in *M. tuberculosis* is up-regulated during acid stress response, and Bansal et al. (2017) demonstrated that PhoP interacts with acid-inducible extra-cytoplasmic SigE to regulate a complex transcriptional of genes. In this study, we observed that three sigma factors (RTCIAT899_RS15835, RTCIAT899_RS13855, and RTCIAT899_RS12250) were induced during the acid stress response. These sigma factors are possibly responsible for regulating other genes important for growth under acid conditions. In addition, forty hypothetical proteins were differentially expressed in acidic conditions (see Table 4 and Supplementary Table S1). Further studies are required to explore the functions of these proteins under acid stress. We also specifically looked at the transcriptome data of orthologues of genes that had been reported to contribute to the acid stress response in other bacteria. For example, the gene glutathione synthase (RTCIAT899_RS01980) and the molecular chaperone DnaK (RTCIAT899_RS00760), have a log_2_FC of 1 and 1.8 respectively. Genes such as *lpiA* (RTCIAT899_RS14615) and *atvA* (RTCIAT899_RS14610) also have a similar log_2_FC, and most of the orthologues of genes involved in the acid response in other bacteria have very low log_2_FC values or are even repressed (Table 5). To our surprise only a very small number of genes was induced in response to 45 min of acid shock. We had designed our experiment based on the article published by Hellweg et al. (2009). Maybe the transcription induction responding to acid stress is slower in *R. tropici*, but there is also the possibility that pH 4.5 is not sufficiently stressful for the bacteria to induce a major short-term response (Figure 6). Although *R. tropici* grows slower at pH 4.5 than at pH 6.8, there is no clear adaptation phase visible in the growth curve obtained at the lower pH.

**Table 4:** List of differentially expressed genes showing the strongest induction or repression.

**Locus tag**	**Location**	**Log2FC**	**COG**	**Function**
**UP-REGULATED GENES pH 4.5 vs. pH 6.8**				
RTCIAT899_RS04615	Chromosome	5.7363999	P	Mn/Zn ABC transporter permease
RTCIAT899_RS04620	Chromosome	5.5273566	P	Mn/Zn ABC transporter ATP-binding protein
RTCIAT899_RS26115	pRtrCIAT899c	5.2777315	P	Potassium-transporting ATPase subunit A
RTCIAT899_RS27660	pRtrCIAT899c	5.1789806	Q	L-lysine 6-monooxygenase (Lysine 6-N-hydroxylase)(Lysine N(6)-hydroxylase) (Lysine-N-oxygenase)
**RTCIAT899_RS10390**	**Chromosome**	**5.1308622**	**P**	**Zinc ABC transporter substrate-binding protein**
RTCIAT899_RS07330	Chromosome	5.1206373	J	30S ribosomal protein S7
RTCIAT899_RS07325	Chromosome	5.0474634	J	30S ribosomal protein S12
**RTCIAT899_RS28825**	**pRtrCIAT899c**	**4.8717347**	**KT**	**Phage shock protein A, PspA**
RTCIAT899_RS13555	Chromosome	4.7818776	P	HmuS-like siderophore transporter
RTCIAT899_RS01890	Chromosome	4.7545879	S	Metallopeptidase
RTCIAT899_RS13560	Chromosome	4.7083112	P	Hemin ABC transporter substrate-binding protein
RTCIAT899_RS07335	Chromosome	4.6010438	J	Elongation factor G
RTCIAT899_RS28820	pRtrCIAT899c	4.464704	S	Hypothetical protein
RTCIAT899_RS29995	pRtrCIAT899c	4.3322649	KG	DeoR family transcriptional regulator
RTCIAT899_RS12735	Chromosome	4.3314229	E	2-isopropylmalate synthase
**RTCIAT899_RS10890**	**Chromosome**	**4.252498**	**EH**	**Acetolactate synthase 3 large subunit**
RTCIAT899_RS04610	Chromosome	4.1803897	P	Mn/Zn ABC transporter substrate-binding protein
RTCIAT899_RS23310	Chromosome	4.173639		Hypothetical protein
RTCIAT899_RS16580	Chromosome	2.2041402	C	F0F1 ATP synthase subunit epsilon
**RTCIAT899_RS16685**	**Chromosome**	**1.8911647**	**C**	**Dihydrolipoamide succinyltransferase**
**DOWN-REGULATED GENES pH 4.5 vs. pH 6.8**				
RTCIAT899_RS11180	Chromosome	−0.7724538	D	Cell division protein FtsZ
RTCIAT899_RS25455	pRtrCIAT899c	−1.0066353	C	Oxidoreductase alpha (molybdopterin) subunit
RTCIAT899_RS15135	Chromosome	−1.0153571	G	Maltodextrin phosphorylase
RTCIAT899_RS02985	Chromosome	−1.2719958	E	Glutamine synthetase 2
RTCIAT899_RS15110	Chromosome	−1.7130873	S	Transglutaminase
RTCIAT899_RS13620	Chromosome	−2.014387	E	Zn-dependent hydrolase
RTCIAT899_RS01510	Chromosome	−2.4492838	G	Sugar ABC transporter substrate-binding protein
RTCIAT899_RS28315	pRtrCIAT899c	−2.5649069	T	Hybrid sensor histidine kinase/response regulator
RTCIAT899_RS28150	pRtrCIAT899c	−2.594403	G	Amino acid ABC transporter substrate-binding protein
RTCIAT899_RS25690	pRtrCIAT899c	−2.6742957	J	Amidase
**UP-REGULATED GENES SHOCK BY ACID (pH 4.5/45 MIN)**				
RTCIAT899_RS24705	pRtrCIAT899c	5.280397	P	Nitrate ABC transporter, permease protein
**RTCIAT899_RS10390**	**Chromosome**	**5.225411**	**P**	**Zinc ABC transporter substrate-binding protein**
RTCIAT899_RS24685	pRtrCIAT899c	4.829516	C	Nitrate reductase
**RTCIAT899_RS28825**	**pRtrCIAT899c**	**4.419892**	**T**	**Phage shock protein A, PspA**
RTCIAT899_RS02060	Chromosome	3.059133	P	Choline-sulfatase
**RTCIAT899_RS10890**	**Chromosome**	**2.250506**	**H**	**Acetolactate synthase 3 large subunit**
**RTCIAT899_RS16685**	**Chromosome**	**1.982522**	**C**	**Dihydrolipoamide succinyltransferase**

*Genes differentially expressed in both conditions are highlighted in boldface. DEGs were those with a p-value in hypergeometrical test inferior to 0.15*.

**Table 5:** Expression of genes whose orthologues are reported to be involved in acid stress response in other bacteria.

**Gene**	**Locus tag**	**log2FC**		**Product**	**References**
		**AB**	**AC**		
*lpiA*	RTCIAT899_RS14615	0.72083386	0.17611909	Low pH inducible protein	Vinuesa et al., 2003
*atvA*	RTCIAT899_RS14610	0.81060257	0.31216314	Acid tolerance and virulence protein	
*olsC*	RTCIAT899_RS13445	−0.16562978	−0.35630822	Ornithine lipid biosynthesis protein OlsC	Vences-Guzmán et al., 2011
*eptA*	RTCIAT899_RS13365	0.45261766	−0.28158652	Phosphoethanolamine transferase	Martinić et al., 2011
*cfa1*	RTCIAT899_RS04265	0.52083757	−0.09048503	Class I SAM-dependent methyltransferase	Shabala and Ross, 2008
*cfa2*	RTCIAT899_RS17235	0.03053786	−0.92576353	Class I SAM-dependent methyltransferase	
*cfa3*	RTCIAT899_RS24775	−0.7897075	−0.08312349	Class I SAM-dependent methyltransferase	
*cfa4*	RTCIAT899_RS18940	0.67863217	0.54558424	SAM-dependent methyltransferase	
*cfa5*	RTCIAT899_RS18960	0.18275374	0.45288988	SAM-dependent methyltransferase	
*Exo*	RTCIAT899_RS06570	−1.33771965	−0.11074631	EPS transporter family	Yuan et al., 2008
*exoX*	RTCIAT899_RS29115	0.10549587	0.69617043	EPS production repressor protein ExoX	Hellweg et al., 2009
*Exo*	RTCIAT899_RS29675	0.33066767	0.12072731	EPS synthesis protein	Cunningham and Munns, 1984
*Exo*	RTCIAT899_RS24840	−1.07127212	−0.35927437	Putative EPS biosynthesis protein	
*exoZ*	RTCIAT899_RS07595	−0.09169763	0.17806745	EPS production protein exoz	
*Exo*	RTCIAT899_RS10240	0.06877117	−0.05075594	EPS biosynthesis protein	
*exoQ*	RTCIAT899_RS29130	−0.87644662	−0.09605896	EPS polymerase ExoQ	
*Exo*	RTCIAT899_RS27545	−0.72836468	0.54432119	EPS polymerization protein	
*Exo*	RTCIAT899_RS24315	−0.11007681	−0.08717239	EPS biosynthesis protein	
*Exo*	RTCIAT899_RS24280	−0.29950943	−0.15973563	EPS biosynthesis protein	
*trkD*	RTCIAT899_RS04105	0.2878656	−0.04007632	Potassium transporter Kup	Foster, 2004
*gshB*	RTCIAT899_RS01980	1.00662168	0.28522761	Glutathione synthase	Riccillo et al., 2000
*kefB/kefC*	RTCIAT899_RS04005	−0.13325459	0.09956478	System protein KefB/KefC	
*Clc*	RTCIAT899_RS10520	−0.01091242	0.05697754	Chloride channel protein	Accardi and Miller, 2004
*Clc*	RTCIAT899_RS04900	−0.00017649	−0.31726611	ClC-type chloride channel	
*Clc*	RTCIAT899_RS14650	0.02874732	−0.2712195	ClC-type chloride channel	
*sycA*	RTCIAT899_RS13450	0.00631424	−0.32276777	ClC-type chloride channel	Rojas-Jiménez et al., 2005
*dnaK*	RTCIAT899_RS00760	1.83544366	0.34592161	Molecular chaperone DnaK	Teixeira-Gomes et al., 2000; Bearson et al., 2006
*groL*	RTCIAT899_RS03935	0.61461457	−0.3681504	Molecular chaperone GroEL	Zanotti and Cendron, 2010
*groS*	RTCIAT899_RS03940	0.9051802	−0.26609308	Molecular chaperone GroES	Zanotti and Cendron, 2010
*ureC*	RTCIAT899_RS13685	−0.75813131	−0.1082779	Urease subunit alpha	Mobley et al., 1995; Bandara et al., 2007
*ureE*	RTCIAT899_RS13675	−0.90771714	0.23427801	Urease accessory protein UreE	
*ureF*	RTCIAT899_RS13670	−0.71555469	−0.03317185	Urease accessory protein UreF	
*ure*	RTCIAT899_RS13700	−1.03377163	−0.10253492	Urease subunit beta	
*ure*	RTCIAT899_RS13715	−1.18510604	0.08358709	Urease accessory protein	
*ure*	RTCIAT899_RS13710	−0.99533936	−0.03468376	Urease subunit gamma	
*ureG*	RTCIAT899_RS13665	−2.47268777	0.08669492	Urease accessory protein UreG	
*ure*	RTCIAT899_RS13690	−0.77758167	0.04132216	Urease accessory protein	
*ure*	RTCIAT899_RS13705	−0.92906417	−0.10094011	Hypothetical protein	

As expected, both, the Tn*5* mutagenesis and RNA-seq approach, produced complementary sets of results. But, the use of both approaches allows the identification of a wider range of genes involved in the acid stress response. Therefore, with this work, we make an important step toward comprehending the genetic bases to understanding the acid stress response in *R. tropici* CIAT899. Much works remains to be done to understand how all the identified genes contribute to the acid stress response. This is especially interesting in the case of genes where the correlation to acid stress is not completely obvious.

## Author contributions

JG-C and CS designed the study. JG-C and CS carried out the experiments. JG-C and LL carried out the data analysis. JG-C and CS were involved in drafting the manuscript and all authors read and approved the final manuscript.

### Conflict of interest statement

The authors declare that the research was conducted in the absence of any commercial or financial relationships that could be construed as a potential conflict of interest.

## Supplementary material

Supplementary Table S1 Complete list of differentially expressed genes. DEGs over-regulated in acid stress conditions. AR_vs_BR_UP (pH 6.8 vs. pH 4.5), AR_vs_BR_DOWN (pH 6.8 vs. pH 4.5) and AR_vs_CR_UP (pH 6.8 vs. acid shock 45 min). Genes differentially expressed in both conditions are highlighted in boldface.

Supplementary Figure S1 Genomic context of Tn5 mutants from *R. tropici* CIAT899. White arrows represent the genes that were interrupted by the transposon Tn*5* (yellow triangles), gray arrows represent neighboring genes to each mutated gene, black lines indicate the genome of *R. tropici* CIAT899.
