# Supplementary figures and images for: Dissecting the Acid Stress Response of Rhizobium tropici CIAT 899
Source: Front Microbiol. 2018 Apr 30;9:846. doi: 10.3389/fmicb.2018.00846 (PMC5936775; doi:10.3389/fmicb.2018.00846)

Genomic context of Tn5 mutants from *R. tropici* CIAT 899

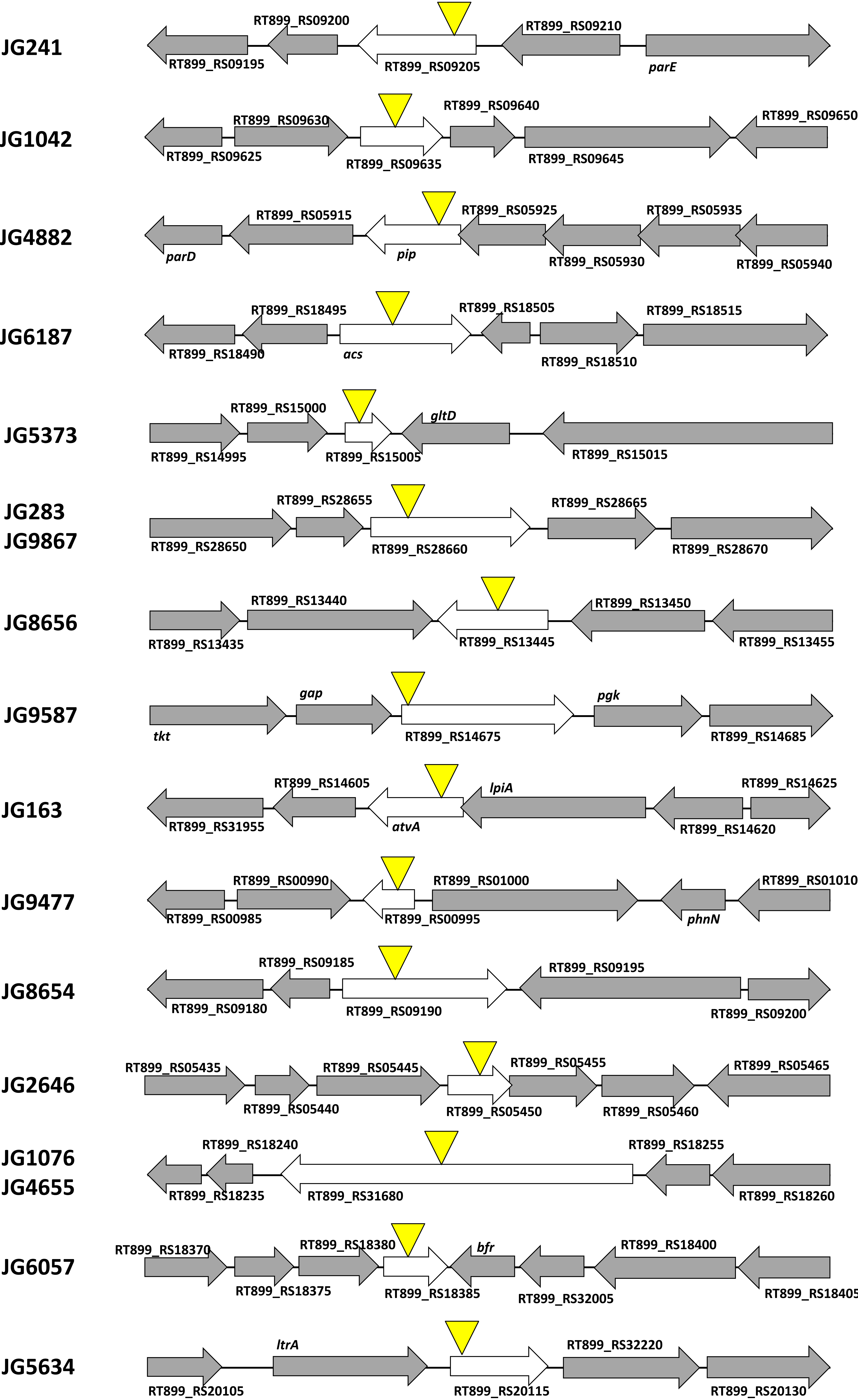

Supplement: Supplementary Figure S1 — Genomic context of Tn5 mutants from R. tropici CIAT899. White arrows represent the genes that were interrupted by the transposon Tn5 (yellow triangles), gray arrows represent neighboring genes to each mutated gene, black lines indicate the genome of R. tropici CIAT899. [file Image_1.PDF]
